# A retrospective cohort study on the impact of gonadotropin dose on embryo quality and pregnancy outcome: identification of optimal dose range

**DOI:** 10.3389/fmed.2026.1820329

**Published:** 2026-06-30

**Authors:** Run Zhao, Linlin Che, Xiao Li, Xiaofang Li, Qianyan Song

**Affiliations:** Department of Reproductive Medicine, Affiliated Hospital of Southwest Medical University Luzhou, Sichuan, China

**Keywords:** antagonist regimen, embryo condition, gonadotropin dose, pregnancy outcome, restricted cubic spline curve

## Abstract

**Objective:**

The study investigates the impact of gonadotropin (Gn) dose on embryo quality and pregnancy outcome in patients receiving controlled ovarian stimulation (COS) with antagonist regimen fertility treatment to identify the optimal dose range of Gn.

**Methods:**

In total, 1854 patients treated for *in vitro* fertilization (IVF) in a single reproductive medicine center between January 2019 and October 2024 were analyzed retrospectively. Patients were categorized into four groups according to the quartile of Gn dose, and general data, embryo quality, and pregnancy outcomes were compared.

**Results:**

The rates of excellent embryos, single blastocyst transfer, clinical pregnancy, and live births were significantly higher and abortion rates were significantly lower for group 1 than for groups 2, 3, and 4. The rates of blastocyst formation were higher in groups 1 and 2 than in groups 3 and 4. Regression analysis showed that total Gn was an independent risk factor for rates of positive human chorionic gonadotropin (HCG), abortion, and live birth and was negatively correlated with blastocyst formation rate (*p* < 0.05). Restricted cubic spline (RCS) curve analysis showed a linear relationship for blastocyst formation, HCG positive, and live birth rates (*p* < 0.05). Increases in Gn above the respective threshold values of 2192.6262, 2395.774, and 1897.138 IU resulted in decreased rates of blastocyst formation, positive HCG, and live birth.

**Conclusion:**

Gn dose influences embryo quality and pregnancy outcome during COS. Adjustment of Gn dose may improve IVF outcomes.

## 1 Introduction

The incidence of infertility in China is approximately 18% (1), and assisted reproductive technology (ART) has helped many infertile couples to produce offspring. Controlled ovarian stimulation (COS), brought about by gonadotropin (Gn) treatment, is a facet of ART that is used to improve the *in vitro* fertilization-embryo transfer (IVF-ET) success rate (2). A Gn dose has been found to impact IVF-ET outcomes, with too low a dose causing follicles to develop unevenly in size and insufficient in maturity, and reducing available egg numbers and embryo formation (3). A high dose of Gn may increase the risk of ovarian hyperstimulation syndrome with rejection of the transplant and detrimental impact on egg quality and endometrial status (4, 5). An appropriate Gn dose may result in a greater number of high-quality eggs and a favorable hormone microenvironment and endometrial state, ensuring a better pregnancy outcome (6). Most earlier studies have focused on the effect of Gn dose on embryo quality and pregnancy outcomes, and few have addressed the Gn dose reference range during COS, resulting in no recommended reference range. The current study explores the relationship between Gn dose and embryo quality and pregnancy outcome in infertile patients treated with COS plus antagonist regimen. Dose–response relationships were analyzed using a restricted cubic spline (RCS) curve to predict the optimal Gn dose range for clinical reference.

## 2 Materials and methods

### 2.1 Participants

The study’s inception date was September 2025. In total, 1854 patients who received IVF in the reproductive medicine center between January 2019 and October 2024 were enrolled. Patients were categorized into four groups by Gn dose quartile: group 1 with Gn dose ≤1,550 IU (*n* = 464), group 2 with 1,550 IU < Gn dose ≤2,194 IU (*n* = 463), group 3 with 2,194 IU < Gn dose ≤2,700 IU (*n* = 493), and group 4 with Gn dose >2,700 IU (*n* = 434). Inclusion criteria were (1) antagonist regimen given for ovulation induction, (2) complete clinical data available, (3) diagnosis of tubal infertility, (4) age ≤40 years old with normal ovarian reserve function, (5) no serious organic lesions, and (6) no immune disease. Exclusion criteria were (1) chromosomal abnormalities in each parent; (2) history of repeated implantation failure; (3) presence of endometriosis, adenomyosis, or uterine fibroids; (4) presence of endometrial polyps or intrauterine adhesions; (5) presence of uterine malformation; and (6) history of recurrent abortion. Ethical approval was granted by the Ethics Committee of the hospital (approval number: KY2024292).

## 3 Research methods

### 3.1 Antagonist ovulation induction protocol

Ovulation induction was initiated on days 2–3 of menstruation, based on patient anti-Mullerian hormone (AMH), antral follicle count (AFC), age, body mass index (BMI), and previous ovarian response. The Gn initiation dose was given at a concentration of follicle-stimulating hormone (FSH) of 100–300 IU (7, 8). The antagonist fixed regimen was given from day 5 or 6 of Gn use but with variationsup to the trigger day. Antagonist initiation was timed based on follicle size and hormone level. The dominant follicle diameter was assessed to be 14–15 mm or >12 mm, and serum estradiol was >300 ng/L (1 ng/L = 3.672 pmoL/L). Triggering was initiated when three dominant follicles ≥17 mm in diameter or two dominant follicles ≥18 mm in diameter were present, and eggs were retrieved 35–37 h later (9). Luteal support was given from retrieval day (10) and consisted of oral dydrogesterone tablets (10 mg tid, Dufton, Abbott, Netherlands) plus intramuscular progesterone injection (60 mg qd, Zhejiang Xianju) or oral dydrogesterone tablets (10 mg tid) plus vaginal progesterone sustained release gel (90 mg qd, Schnaughton, Germany, Merck Serono Co., Ltd.). Cleavage embryo transfer was performed on the third day after oocyte retrieval; blastocyst transfer was performed on the fifth day after oocyte retrieval, and luteal support continued after embryo transfer.

### 3.2 Embryo culture and transfer

IVF was achieved via intracytoplasmic sperm injection (ICSI) and observed 16–18 h after insemination with cleavage on the third day. Embryos were scored at cleavage, according to the standards mentioned in Assisted Reproduction Laboratory Technology (edited by Huang Guoning) and guidelines of the Society for Assisted Reproductive Technology (SART, 2010; Alpha Executive and ESHRE, 2011). After 5 days of culture, developed blastocysts were scored according to the blastocyst scoring standard (11) based on the Gardner scoring system (12), and high-quality blastocysts with grade 4BB or above were considered appropriate for embryo development. Embryos were transferred, as appropriate to patient age and IVF indications, with the remaining embryos frozen and stored.

### 3.3 Embryo status and pregnancy outcome criteria

Definitions used in the study:

- Metaphase II (MII) ovum rate = MII ovum number/total retrieved ovum number × 100%; usable embryo = usable embryo number/2PN cleavage number × 100%;
- D3 high-quality embryo rate = D3 high-quality embryo number/2PN cleavage number × 100%;
- Blastocyst formation rate = blastocyst number/blastocyst culture number ×100%.
- HCG positive rate = number of HCG-positive patients/the number of transplant cycle patients ×100%. Patients were considered to be HCG positive when serum human chorionic gonadotropin (*β*-hCG) > 5 IU/L was detected 2 weeks after transplantation;
- Clinical pregnancy rate = the number of patients with clinical pregnancy/the number of patients in the transplant cycle×100%. Clinical pregnancy was considered to have occurred when the pregnancy sac (intrauterine or extrauterine) could be detected by vaginal ultrasound 4–5 weeks after transplantation;
- Abortion rate = (number of early abortion cycles + number of late abortion cycles)/number of clinical pregnancy cycles. Abortion was due to pregnancy termination resulting from an inviable embryo or fetus;
- Live birth rate = number of live birth cycles/number of transplant cycles.

## 4 Statistical analyses

SPSS 26.0 software and R language (Version 4.5.1) were used for data analysis. Qualitative data are presented as numbers or percentages, and measurement data are presented as mean ± standard deviation (*x ± s*). The *χ*^2^ test was used to analyze qualitative data, whereas ANOVA was used to compare measurement data. RCS curves were used to analyze dose–response relationships between Gn, embryo condition, and pregnancy outcome, as well as to predict an optimal Gn dose range. A *p*-value <0.05 was considered to indicate statistical significance.

## 5 Results

### 5.1 Comparison of general condition, embryo quality, and pregnancy outcome

The mean maternal age of group 1 patients was significantly lower than that of any other group, and that of group 2 was lower than that of groups 3 and 4. In addition, the mean BMI was lower in group 1 than in groups 3 or 4, and that of groups 2 and 3 was lower than that of group 4. Basal FSH was lower for group 1 patients than for other groups, and AMH was higher. Differences in embryo and pregnancy indicators were found, with higher rates of embryo and single blastocyst transfers for group 1 patients on D3 than for any other group. Blastocyst formation was at higher rates for groups 1 and 2 than for groups 3 and 4. Clinical pregnancy and live birth rates were also higher and abortion rates were lower for Group 1 than for Group 4. No statistically significant differences were found in other general conditions or pregnancy outcomes among the four groups (Table 1).

**Table 1:** Comparison of general conditions, embryo quality, and pregnancy outcomes of the four groups of patients.

Category	Group 1 (Gn dose ≤ 1,550)	Group 2 (1,550 < Gn dose ≤ 2,194)	Group 3 (2,194 < Gn dose ≤ 2,700)	Group 4 (Gn dose> 2,700)	*P*
Number of cycles	464	463	493	434	
Infertility type					
Primary infertility	252 (54.3%)	223 (48.2%)	235 (47.7%)	198 (45.7%)	0.056
Secondary infertility	212 (45.7%)	240 (51.8%)	258 (52.3%)	235 (54.3%)	
The woman’s age (years)	28.98 ± 3.51	30.12 ± 3.90	32.03 ± 4.04	32.97 ± 4.26	<0.001
BMI (kg/m^2^)	22.07 ± 3.16	22.64 ± 3.36	23.06 ± 3.49	23.68 ± 3.70	<0.001
Basic FSH(mIU/ml)	7.68 ± 1.85	8.22 ± 1.84	8.56 ± 2.06	8.77 ± 2.38	<0.001
AMH(ng/ml)	6.66 ± 4.83	4.66 ± 3.60	3.87 ± 3.12	3.18 ± 2.37	<0.001
Endometrial thickness on day of transplantation (mm)	11.13 ± 2.43	11.27 ± 2.36	11.36 ± 2.64	10.93 ± 2.62	0.335
MII oocyte rate(%)	87.48 ± 16.28	89.26 ± 24.22	89.21 ± 14.70	89.87 ± 15.65	0.219
Embryo utilization rate of D3(%)	60.11 ± 24.57	61.57 ± 25.13	59.61 ± 27.24	58.95 ± 26.37	0.473
D3 High-quality embryo rate(%)	56.56 ± 27.68	52.93 ± 30.14	50.77 ± 30.37	52.08 ± 30.82	0.021
Blastocyst formation rate(%)	70.70 ± 22.24	70.69 ± 22.95	62.50 ± 27.09	63.00 ± 26.31	<0.001
Single cleavage embryo transfer rate (%)	27 (14.7%)	42 (19.4%)	43 (18.1%)	35 (18.4%)	0.637
Double cleavage embryo transfer rate (%)	110 (59.8%)	139 (64.4%)	158 (66.7%)	127 (66.8%)	0.435
Single blastocyst transfer rate (%)	45 (24.5%)	30 (13.9%)	31 (13.1%)	21 (11.1%)	0.001
Double blastocyst transfer rate (%)	2 (1.1%)	5 (2.3%)	5 (2.1%)	7 (3.7%)	0.413
Quality embryo transfer rate (%)	156 (84.8%)	179 (82.9%)	198 (83.5%)	158 (83.2%)	0.961
HCG positive rate (%)	110 (59.8%)	117 (54.2%)	128 (54.0%)	89 (46.8%)	0.095
Clinical pregnancy rate (%)	90 (48.9%)	83 (38.4%)	91 (38.4%)	67 (35.3%)	0.039
Abortion rate (%)	6 (6.7%)	7 (8.4%)	8 (8.8%)	13 (19.4%)	0.049
Live birth rate (%)	84 (45.7%)	76 (35.2%)	83 (35.0%)	54 (28.4%)	0.006

### 5.2 Impact of Gn dose on pregnancy outcome

Multivariate binary logistic regression analysis was used to correct for confounding factors, allowing analysis of the impact of maternal age, BMI, Gn dose, AMH, and basal FSH on pregnancy outcome. BMI was found to be an independent risk factor for HCG positive and clinical pregnancy rates, and total Gn dose was an independent risk factor for HCG positive (OR = 1.00, 95% CI: 0.999–1.000, *p* = 0.018), miscarriage (OR = 1.00, 95% CI: 1.000–1.001, *p* = 0.047) and live birth rates (OR = 1.00, 95% CI: 1.000–1.000, *p* = 0.013). No significant effect of Gn dose on the clinical pregnancy rate was found (Table 2).

**Table 2:** Impact of Gn dose on pregnancy outcomes.

Category	HCG positive		Clinical pregnancy		Abortion		Live birth	
	OR (95% CI)	*p*	OR (95% CI)	*p*	OR (95% CI)	*p*	OR (95% CI)	*p*
The woman’s age	0.999 (0.965–1.035)	0.975	0.967 (0.933–1.002)	0.062	1.071 (0.980–1.171)	0.132	0.960 (0.926–0.996)	0.028
BMI	1.084 (1.040–1.131)	<0.001	1.050 (1.007–1.094)	0.022	1.049 (0.949–1.159)	0.349	1.039 (0.996–1.084)	0.080
AMH	0.997 (0.951–1.044)	0.882	1.005 (0.960–1.053)	0.818	1.109 (1.004–1.225)	0.042	0.986 (0.941–1.034)	0.569
Basic FSH	1.001 (0.934–1.074)	0.968	1.002 (0.933–1.076)	0.948	1.001 (0.836–1.199)	0.992	1.004 (0.933–1.080)	0.913
Gn dosage	1.000 (0.999–1.000)	0.003	1.000 (1.000–1.000)	0.058	1.000 (1.000–1.001)	0.047	1.000 (1.000–1.000)	0.013

### 5.3 Impact of Gn dose on embryo quality

Linear regression analysis was used to correct for confounding factors, allowing analysis of the impact of maternal age, BMI, Gn dose, AMH, and basal FSH on embryo quality. Gn dose and age were negatively correlated with blastocyst formation rate, and AMH was negatively correlated with D3 available embryo rate. The Gn dose had no significant effect on MII oocyte, D3 available embryo, or D3 high-quality embryo rates (Table 3).

**Table 3:** Impact of Gn dose on embryo quality.

Variables	Unstandardized coefficient		Standardization coefficient	*t*	*p*	Unstandardized coefficient		Standardization coefficient	*t*	*p*	Unstandardized coefficient		Standardization coefficient	*t*	*p*	Unstandardized coefficient		Standardization coefficient	*t*	*p*
	B	Standard error(SE)	*β*			B	Standard error(SE)	*β*			B	Standard error(SE)	*β*			B	Standard error(SE)	*β*		
Intercept	80.807	4.702		17.186	<0.001	67.764	6.709		10.101	<0.001	63.812	7.741		8.244	<0.001	92.689	8.952		10.354	<0.001
female age	0.081	0.107	0.019	0.760	0.447	−0.090	0.153	−0.015	−0.590	0.555	−0.203	0.176	−0.029	−1.154	0.249	−0.525	0.203	−0.086	−2.581	0.010
BMI (kg/m^2^)	0.029	0.127	0.005	0.227	0.821	−0.120	0.181	−0.016	−0.664	0.507	0.144	0.208	0.017	0.692	0.489	−0.126	0.237	−0.017	−0.530	0.596
AMH	0.151	0.118	0.032	1.282	0.200	−0.366	0.168	−0.054	−2.174	0.030	−0.101	0.194	−0.013	−0.522	0.602	−0.109	0.196	−0.019	−0.553	0.580
Basic FSH	0.253	0.213	0.029	1.189	0.234	0.234	0.303	0.019	0.772	0.440	−0.493	0.350	−0.034	−1.408	0.159	0.182	0.419	0.014	0.434	0.664
Gn dosage-	0.001	0.001	0.042	1.570	0.117	−0.001	0.001	−0.033	−1.231	0.219	−0.001	0.001	−0.038	−1.401	0.161	−0.004	0.001	−0.111	−3.055	0.002
dependent variable	MII oocyte rate					Embryo utilization rate of D3					D3 high-quality embryo rate					Blastocyst formation rate				

### 5.4 RCS curve analysis of Gn dose with embryo and pregnancy outcomes

**Figure 1:**
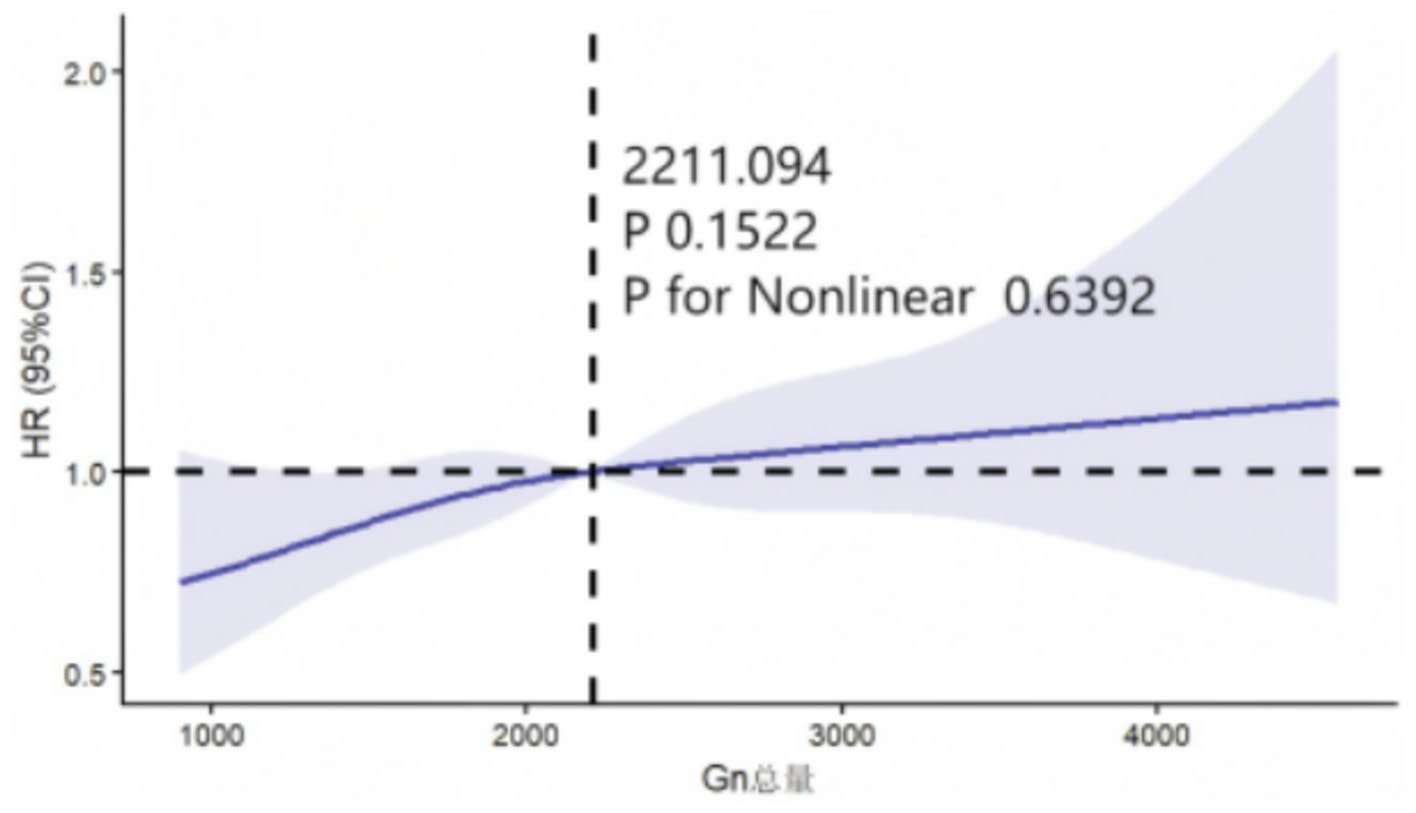
MII oocyte rate.

**Figure 2:**
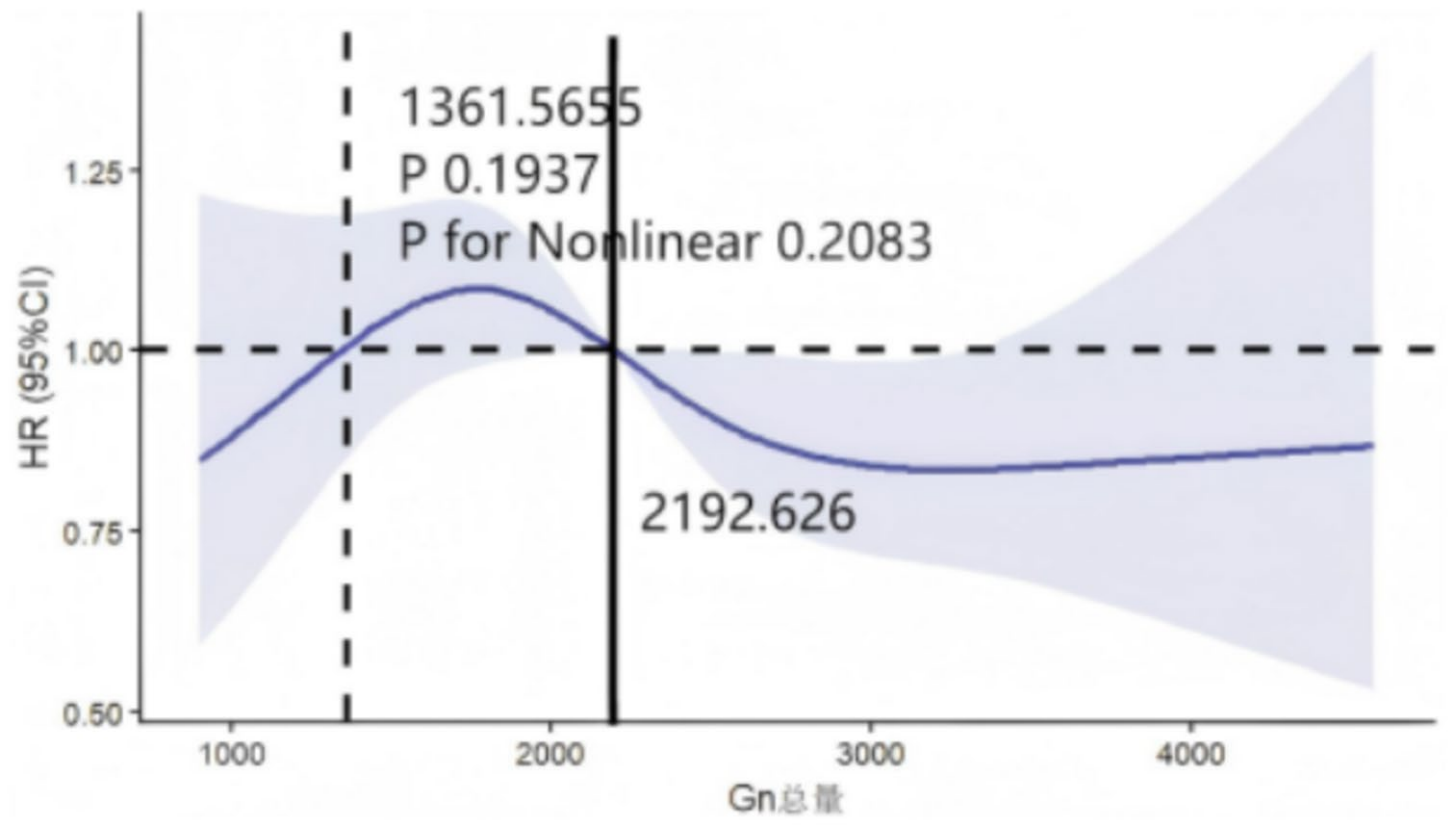
Embryo utilization rate of D3.

**Figure 3:**
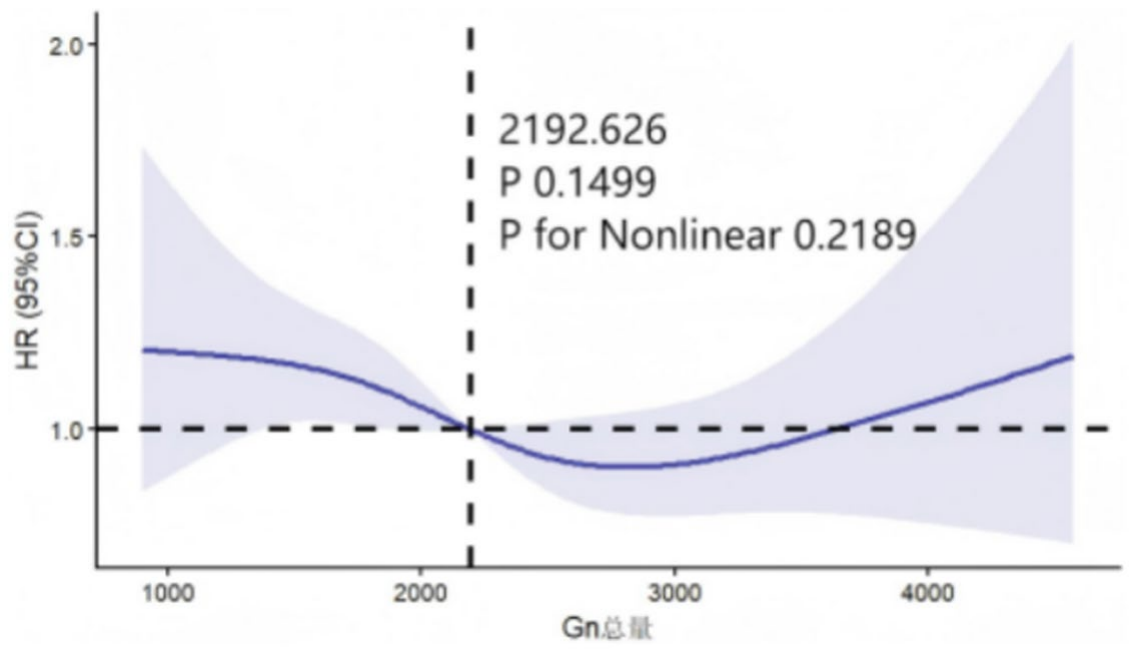
D3 high-quality embryo rate.

**Figure 4:**
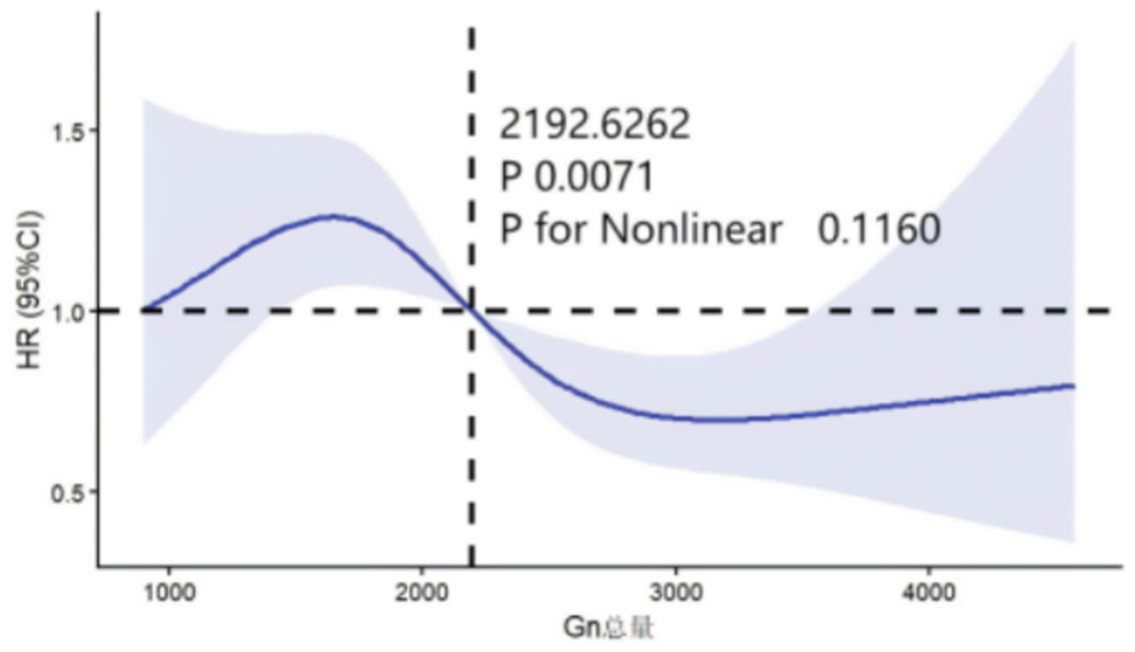
Blastocyst formation rate.

**Figure 5:**
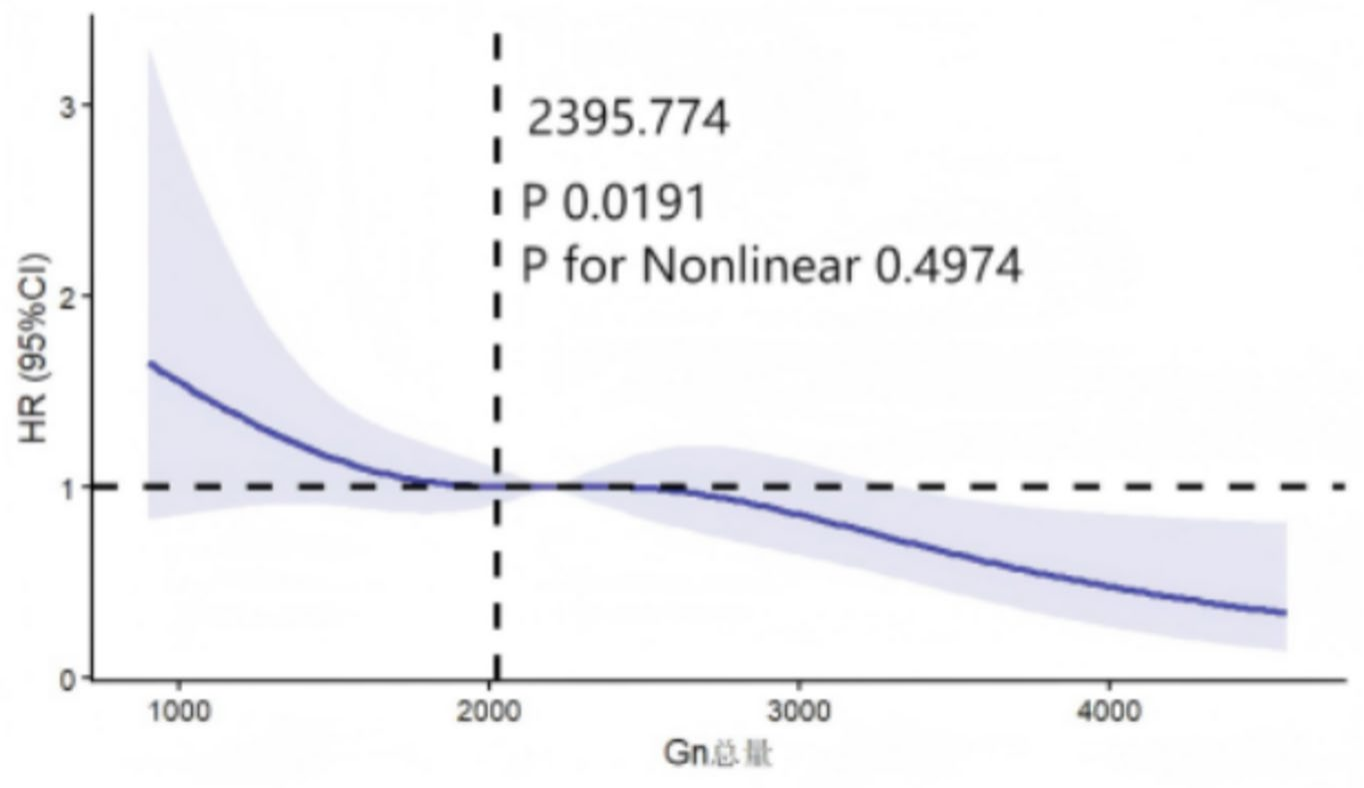
Positive HCG.

**Figure 6:**
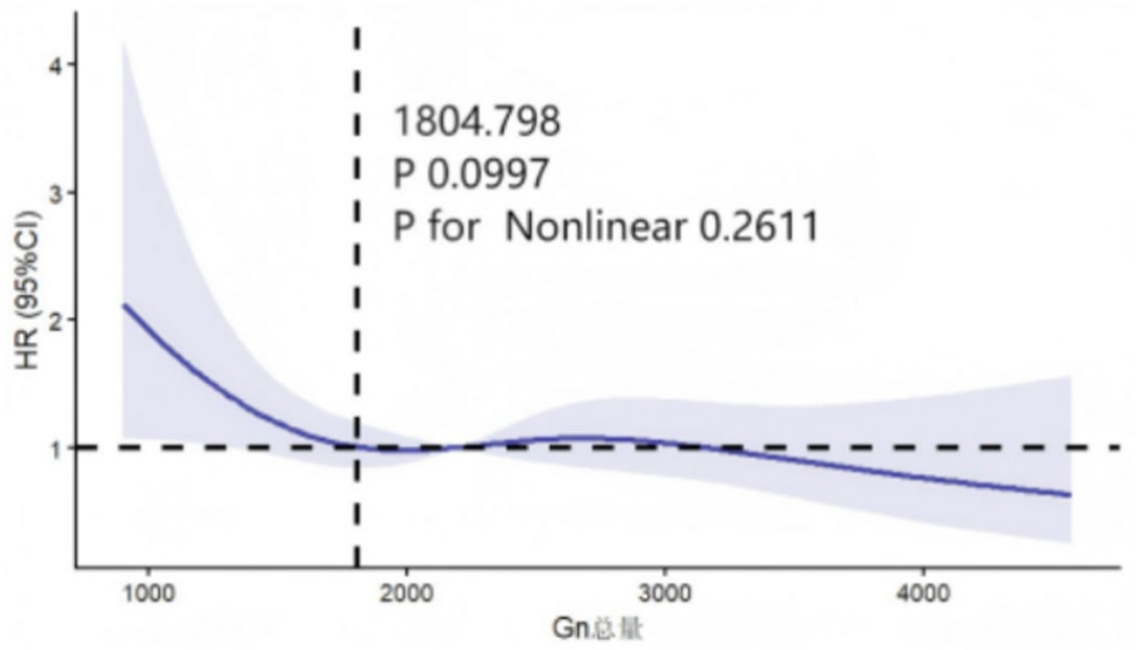
Clinical pregnancy.

**Figure 7:**
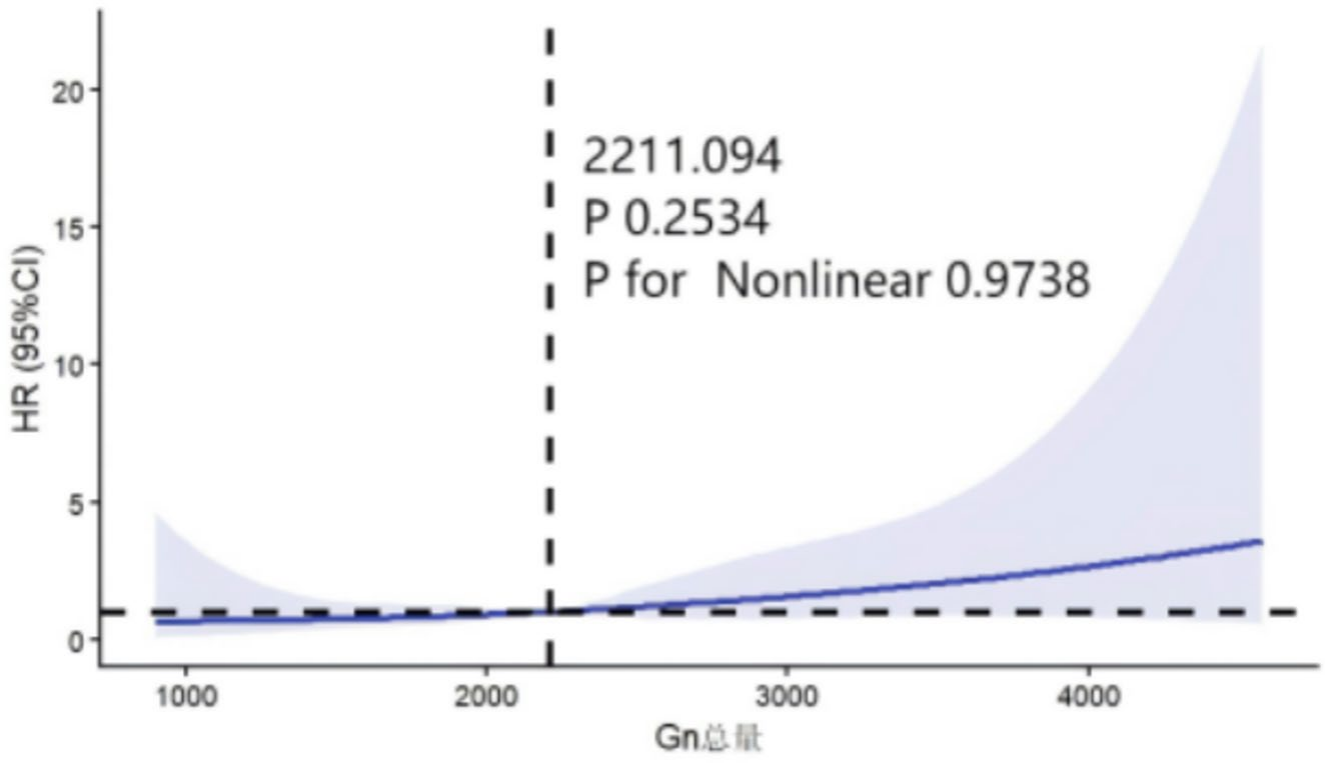
Abortion.

**Figure 8:**
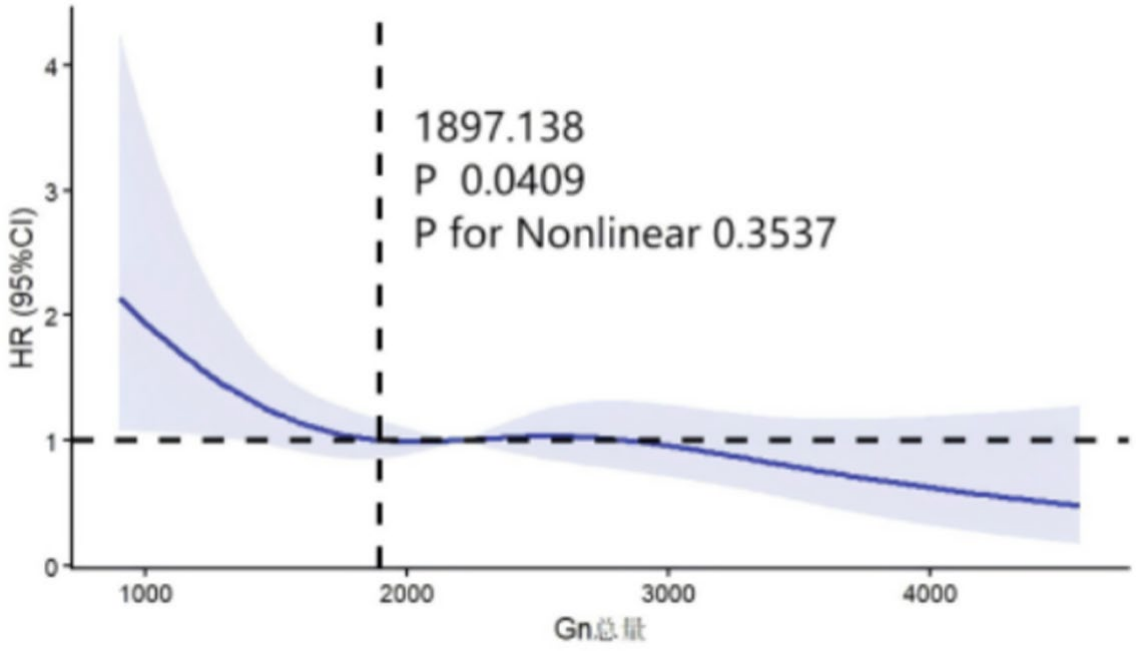
Live births.

Threshold values of AMH = 3.335 ng/mL, maternal age = 31 years, BMI = 22.4 kg/m^2^, and basal FSH = 8.11 mIU/ml were used, at which exogenous Gn dose showed a significant linear relationship with blastocyst formation, HCG positive, and live birth rates. Threshold Gn doses were established, above which blastocyst formation, HCG positive, and live birth rates decreased with increasing Gn doses. The thresholds were 2192.6262 IU for blastocyst formation, 2395.774 IU for HCG positive, and 1897.138 IU for live birth rates (Figures 1–8).

## 6 Discussion

The safety and success rates of ART, of which COS is a key component, have attracted considerable attention. Multiple follicles are stimulated to develop synchronously to produce multiple mature oocytes and improve the success rates of single oocyte retrieval cycles. Successful COS requires the use of exogenous Gns, too high or too low a dose, which may decrease the IVF success rate. However, no recommended reference range for exogenous Gns has been established (13). The current analysis shows that group 1 patients had a higher excellent embryo rate than other groups on D3, blastocyst formation was at higher rates in groups 1 and 2, clinical pregnancy and live birth rates were higher for group 1 than for group 4, and the group 1 abortion rate was significantly lower than for group 4. These findings were consistent with those of Ni et al. (2, 6, 13–18), who also reported that increasing exogenous Gn had an unfavorable impact on high-quality embryo, blastocyst formation, clinical pregnancy, live birth, and abortion rates. Aboulghar et al. (19) found that high doses of exogenous Gns stimulate estrogen concentrations into the supra-physiological range, resulting in oocyte damage and embryo development blockade. In addition, Yang et al. (20) found a positive correlation between exogenous Gn dose and oocyte maturation. Higher doses may stimulate the simultaneous development of multiple follicles during ovulation induction, affecting the natural selection of dominant follicles and resulting in the recruitment of some secondary follicles, which affects embryo quality (21). High doses of exogenous Gns may also interfere with oocyte meiosis and chromosomal separation, increasing the incidence of embryo aneuploidy (22) with consequences for high-quality embryo and blastocyst formation rates. The current study also found better pregnancy outcomes for Group 1 patients despite the much higher rate of single embryo transfer, probably because higher doses of exogenous Gn increased the occurrence of chimerism after embryo transfer in groups 2, 3, and 4. In addition, excessive estrogen levels may have affected endometrial receptivity, resulting in increased abortion and decreased clinical pregnancy and live birth rates (6, 14).

RCS curve analysis of the dose–response relationship between exogenous Gn and embryo quality/pregnancy outcome showed a linear correlation with blastocyst formation, HCG positive, and live birth rates after correction for AMH = 3.335 ng/mL, female age = 31 years old, BMI = 22.4 kg/m^2^, and basal FSH = 8.11 mIU/ml. When the total amount of Gn reaches the threshold, as the total amount of Gn increases, the blastocyst formation rate, the positive rate of HCG, and the live birth rate decrease, the thresholds are 2192.6262 IU, 2395.774 IU, and 1897.138 IU respectively. No significant relationship was found between exogenous Gn dose and MII egg, D3 available embryo, D3 excellent embryo, clinical pregnancy, or abortion rates up to threshold values. Increases above the threshold values of 2192.626 IU for D3 available embryo, 2192.626 IU for D3 excellent embryo, and 1804.798 IU for clinical pregnancy resulted in decreased rates for all these metrics, and above 2211.094 IU for MII egg and 2211.094 IU for abortion increased rates for these two metrics. Baker et al. (2) found that a total Gn dose >3,000 IU resulted in a lower live birth rate but that a total dose 1,000–1999 IU produced a higher live birth rate than a total dose of <1,000 IU. These values differ from the optimal exogenous Gn dose range reported in this study, perhaps due to differences in the test population.

In summary, exogenous Gn doses that are too high or too low were found to affect embryo quality and pregnancy outcome, illustrating the need to control the exogenous Gn dose during ovulation induction to maximize embryo quality and pregnancy outcome. An optimal dose range was found to be 1800–2,400 IU for infertile patients receiving an antagonist regimen to assist during pregnancy. The current study was a retrospective single-center study, and it included only infertile patients receiving antagonist protocols for ovulation induction to avoid the influence of different induction protocols on the results. Patients were divided into four groups based on Gn dosage quartile. Some selection bias and other limitations may be present. Further studies with larger sample sizes, multi-center protocols, and prospective randomized controlled trials with different endometrial preparation protocols are required to verify the current findings and inform measures to improve embryo quality and pregnancy outcomes for infertile patients.

## Data Availability

The original contributions presented in the study are included in the article/Supplementary material, further inquiries can be directed to the corresponding author/s.
